# Activation of liver stromal cells is associated with male-biased liver tumor initiation in *xmrk* and *Myc* transgenic zebrafish

**DOI:** 10.1038/s41598-017-10529-1

**Published:** 2017-09-04

**Authors:** Qiqi Yang, Chuan Yan, Zhiyuan Gong

**Affiliations:** 1 Department of Biological Sciences, National University of Singapore, Singapore, Singapore; 2 National University of Singapore graduate school for integrative sciences and engineering, National University of Singapore, Singapore, Singapore

**Keywords:** Cancer models, Cancer microenvironment

## Abstract

Hepatocellular carcinoma (HCC) is more prevalent in men than in women. Previously we have found that some stromal cells, including hepatic stellate cells (HSCs), neutrophils and macrophages, play crucial roles in promoting sex disparity in *kras* ^*V12*^-induced zebrafish HCC. The activation of HSCs is mediated by serotonin while activation of neutrophils and macrophages is mediated by cortisol. To ensure that these findings are also applicable to other oncogene induced tumors, stromal cell activation was compared between male and female fish during liver tumorigenesis initiated by *xmrk* or *Myc* oncogene. Consistently, we observed male-biased liver tumorigenesis in the *xmrk* and *Myc* models. In both models, there was a higher rate of HSC activation accompanied with a higher level of serotonin in male liver tumors. For tumor-infiltrated neutrophils and macrophages, significantly higher densities in male liver tumors were observed in both *xmrk* and *Myc* models. However, the male-biased increase of cortisol was observed only in *xmrk*- but not apparently in *Myc* expressing liver tumors. Overall, these observations are consistent with the observations in the *kras* liver tumor model, indicating that the serotonin- and cortisol-mediated pathways also play roles in sex disparity of liver tumors caused by other molecular pathways.

## Introduction

Hepatocellular carcinoma occurs more frequently and aggressively in men than in women^1^. Based on the animal model studies, the gender disparity might be owing to a sex hormone related mechanism, with a stimulating role of androgen and an inhibitory role of estrogen^2^. Administration of estrogens inhibits HCC development in diethylnitrosamine (DEN)-treated male mice. On the contrary, ovariectomy or testosterone supplement increase occurrence of HCC in female mice^3^. However, clinical trials targeting sex hormone pathways, e.g. by using the estrogen receptor modulator tamoxifen, synthetic progestin (megestrol) and androgen antagonist flutamide, produced inconclusive results as these treatments in the clinical trials did not show significant improvement^4–7^.

Most of HCC arise on a background of hepatic fibrosis and/or inflammation and it has been increasingly recognized that some stromal cells in the tumor microenvironment (TME) are abnormally activated in both HCC animal models and HCC patients. Hepatic stellate cells (HSCs) are the main matrix-producing cells in the TME and play key roles in the progression of liver fibrosis. Co-culture of HSCs with HCC cells showed upregulated expression of proinflammatory cytokines and proangiogenic genes^8^. Depletion of HSCs from pre-established fibrosis attenuated fibrogenesis in a mouse liver disease model^9^. Co-transplantation of human intratumoral HSCs with HCC into nude mice showed enhanced HCC progression by HSCs and HSC density is correlated with the overall and recurrence-free survival, thus providing promising prognostic biomarkers^10^. Consistent with this, we also reported recently that HSC has a higher density and activation ratio in *kras* ^*V12*^-expresing tumor in the zebrafish^11^.

Tumor associated macrophages (TAMs) and tumor associated neutrophils (TANs) play key roles in hepatic inflammation and cytokines produced by TAMs promote tumor growth, angiogenesis and suppression of adaptive immunity^12^. DEN administration promotes IL-6 production in Kupffer cells in male mice and ablation of IL-6 attenuates liver carcinogenesis in male and abolishes the gender difference^13^. In HCC patients after resection, the number of intratumoral neutrophils is significantly correlated with the early recurrence which could be a poor prognostic factor^14^. In our *kras* ^*V12*^-expresing zebrafish liver tumor model, Infiltrations of TAMs and TANs are significantly higher in male tumors than in female tumors. Pro-tumor genes are also more strongly expressed in the TAMs and TANs of male tumors, correlating to a faster tumor progression^15^.

As we previously reported, the activation of stromal cells has a close correlation with tumor progression, especially HSCs, TAMs and TANs^11, 15, 16^. Interestingly, these stromal cells appear to also contribute to the sex disparity of HCC in the *kras* ^*V12*^ model. In male *kras* ^*V12*^ transgenic zebrafish, a higher level of serotonin activates HSCs and causes accelerated liver tumor progression^11^. Male *kras* ^*V12*^ transgenic zebrafish also produced higher of cortisol to cause enhanced TAN and TAM infiltration to accelerate liver tumor progression^15^. However, whether the importance of these stromal cells is universal to other oncogene-induced tumors or only specific to *kras* ^*V12*^–induced tumors remains unclear. Previously, our laboratory has generated two other oncogene induced HCC models in transgenic zebrafish; one is by inducible expression of *xmrk* oncogene^17^ and the other is inducible expression of the mouse *Myc* oncogene^18^. Both transgenic lines have been generated with the same Tet-on transgenic system where the oncogenes are expressed under the hepatocyte-specific *fabp10a* promoter and the expression was induced only by addition of the chemical inducer, doxycycline. In the present study, we first confirmed the sex disparity in HCC development in these two oncogene transgenic models. Then we found that positive correlations of these stroma cells to the progression of liver tumors in both the *xmrk* and *Myc* transgenic models, including conserved molecular pathways such as serotonin activated HSCs and cortisol enhanced TANs and TAMs. These mechanisms also similarly contribute to sex disparity of liver tumors in the *xmrk* and *Myc* models.

## Results

### Enhanced cell proliferation during hepatocarcinogenesis in male zebrafish with transgenic expression of *xmrk* or *Myc* oncogene

**Figure 1:**
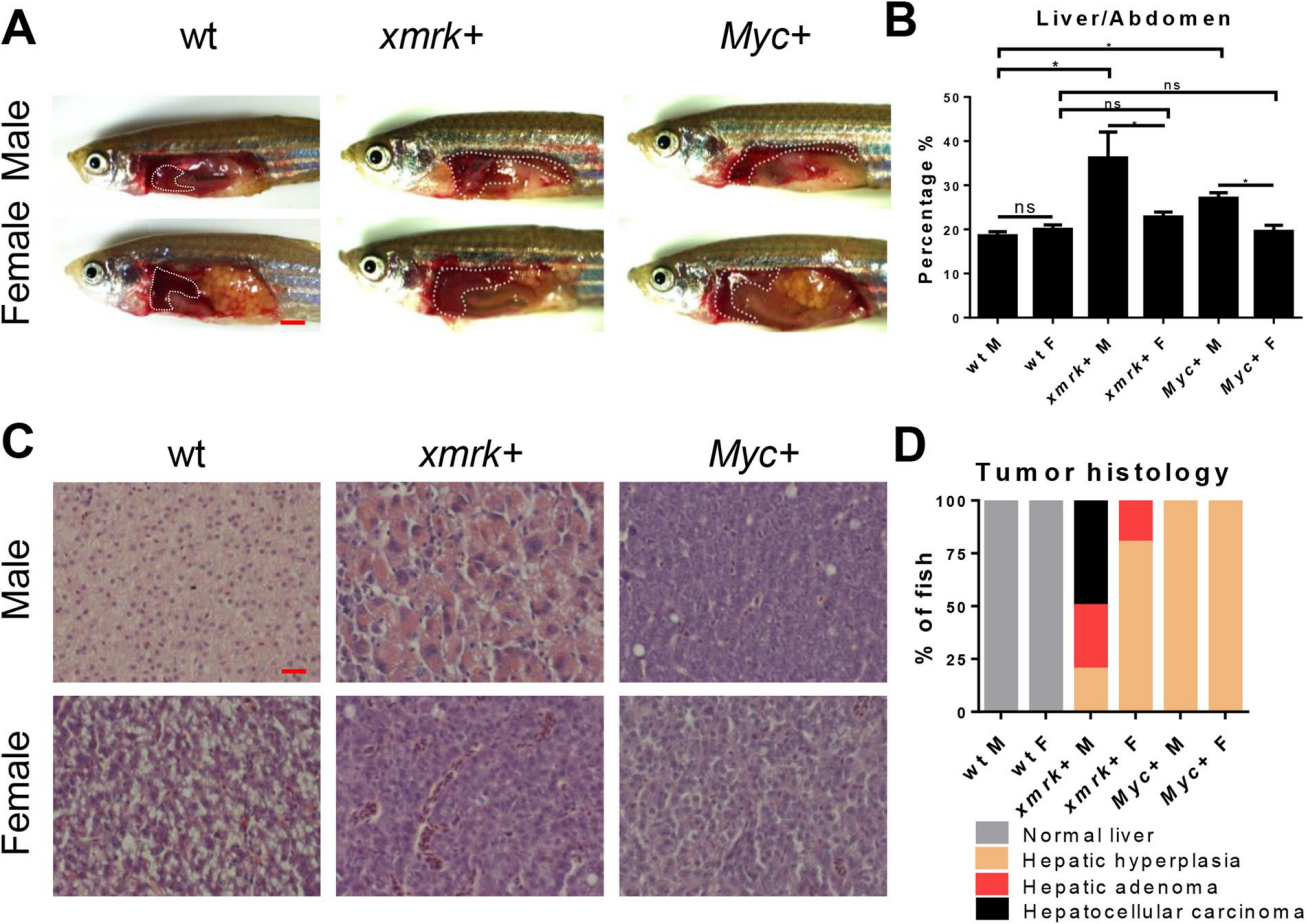
Characterization of sex disparity in *xmrk* and *Myc*-induced HCC progression. Three-month-old, *xmrk*+, *Myc*+ and wildtype zebrafish were treated with 60 μg/ml dox for 7 days. 10 fish were analysed in each group and the experiment was repeated multiple times. (**A**) Gross morphology of male and female fish (left lateral view). The livers are outlined. (**B**) Quantification of percentage of liver area to abdomen area. (**C**) Representative images of liver sections of male and female fish after H&E staining. (**D**) Quantification of tumor histology in male and female fish. *P < 0.05. Scale bars: 2 mm in (**A**) and 20 μm in (**C**).

As we reported previously, *kras*^*V12*^-expressing male tumors shows an accelerated HCC progression^11, 15, 19^. To investigate if *xmrk-* or *Myc*–induced HCC has similar sex disparity, male and female *xmrk*+ or *Myc*+ fish were exposed to doxycycline for 7 days. 2D liver size was measured after the treatment (Fig. 1A). In the *xmrk*+ and *Myc*+ expressing fish of both sexes, male tumor livers were bigger than the female tumor livers in both *xmrk* and *Myc*-expressing fish (Fig. 1B). Histologically, male developed more aggressive tumors than female in *xmrk*-expressing liver. In wildtype livers, the 2-cell hepatic plate was well organized while in *xmrk*-expressing male tumor, oncogenic hepatocytes had prominent and multiple nucleoli and lost the 2-cell plate organization (Fig. 1C). In *xmrk*-expressing female tumors, most of the tumors were at early HCC stage with loss of 2-cell plate and prominent nucleoli. However, in *Myc*-expressing fish, all of the liver tumors were at the hyperplastic stage without apparent sex disparity. As summarized in Fig. 1D, 50% of male *xmrk*-expressing tumor showed carcinoma, 30% showed adenoma and the remaining 20% had hepatic hyperplasia. In contrast, 80% of female *xmrk*-expressing tumor showed hepatic hyperplasia and the remaining 20% had adenoma histology. However, the *Myc*-expressing livers in both female and male had similar hepatic hyperplasia histology.

**Figure 2:**
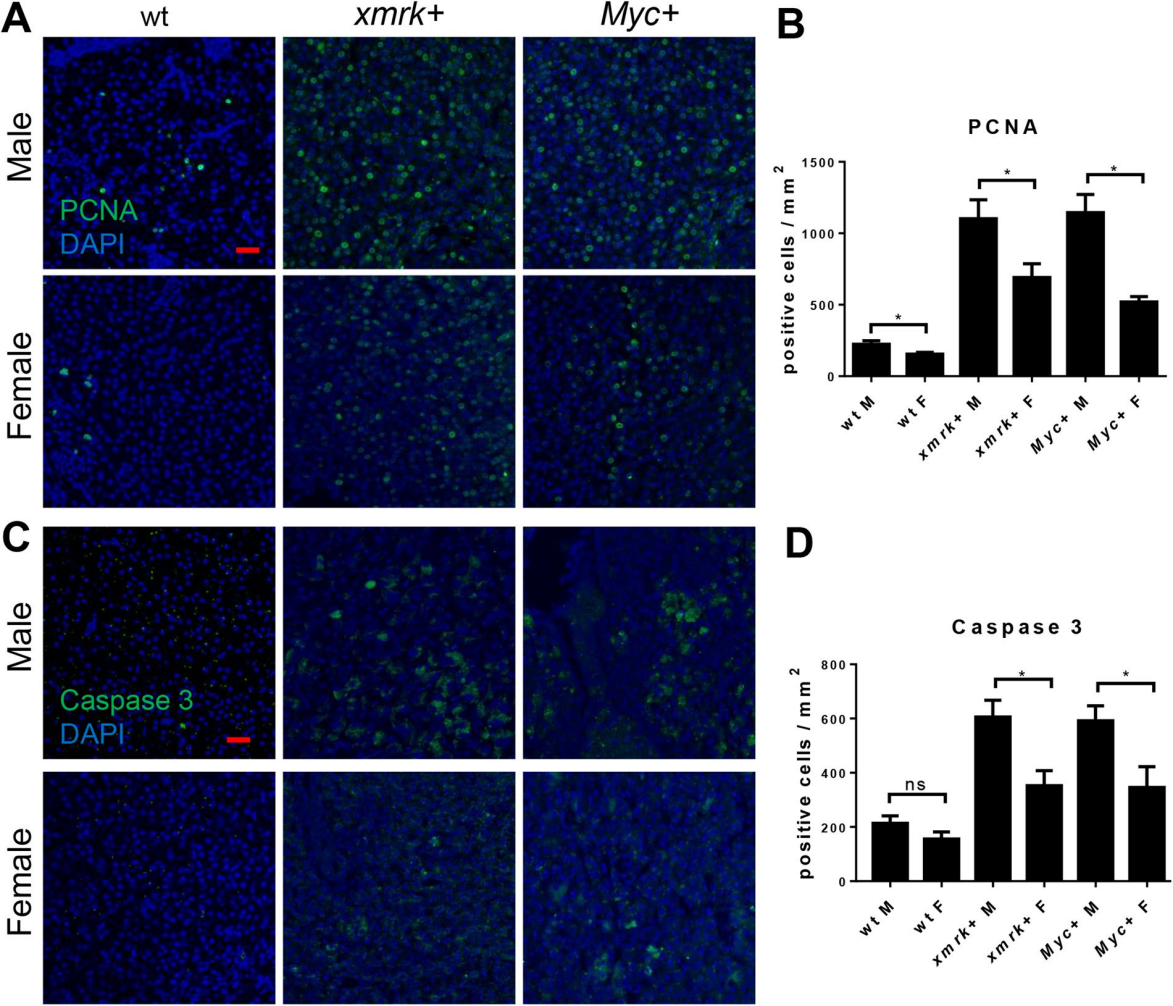
Proliferation and apoptosis in the livers of male and female *xmrk*+ and *Myc*+ fish following oncogene activation. 10 fish were analysed in each group and the experiment was repeated multiple times. Proliferation and apoptosis were examined by PCNA and Caspase 3 staining respectively. (**A**) IF staining of PCNA in liver sections. (**B**) Quantification of densities of proliferating liver cells (PCNA+). (**C**) IF staining of Caspase-3 in liver sections. (**D**) Quantification of densities of apoptotic liver cells (Caspase 3+). *P < 0.05. Scale bars: 20 μm.

To further investigate the molecular mechanism in both transgenic lines, the cell proliferation and apoptosis were examined by immunofluorescence (IF) staining of PCNA and caspase-3, respectively. The proliferating cells were comparable and had a low percentage in both sexes of normal livers of wildtype fish, while *xmrk* and *Myc-*expression promoted hepatocyte proliferation significantly (Fig. 2A,B). Levels of hepatocyte apoptosis had also been accelerated by *xmrk* and *Myc*-expression in both sexes (Fig. 2C,D). In wildtype fish, there were no significant difference in both proliferating cells and apoptotic cells between female and male.

### Correlation of serotonin level, activated HSCs and higher induction of male carcinogenesis

**Figure 3:**
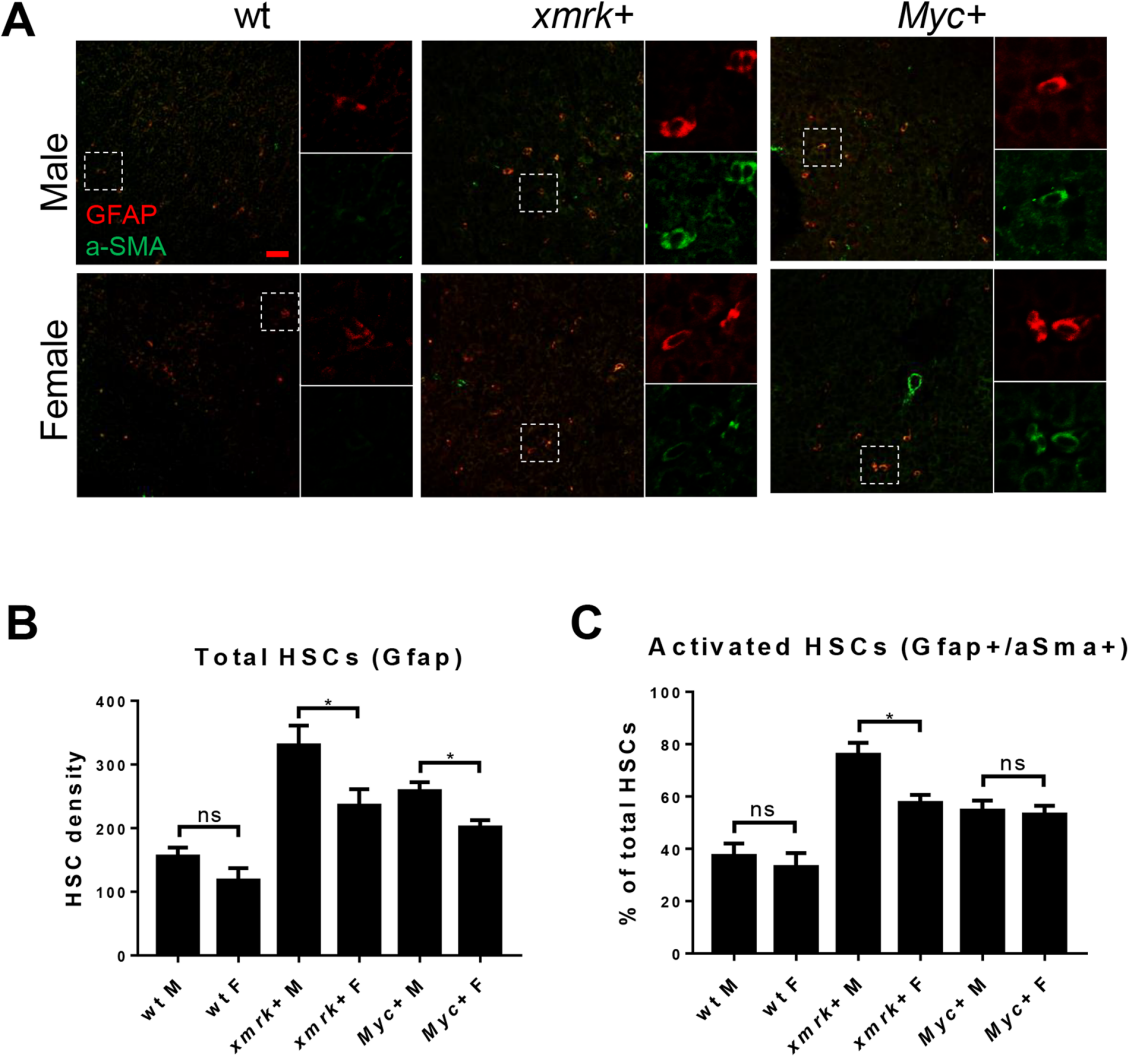
Determination of HSCs and activated HSCs in the livers of male and female *xmrk*+ and *Myc*+ fish following oncogene activation. 10 fish were analyzed in each group and the experiment was repeated once for reproducibility. (**A**) IF co-staining of GFAP (red) and a-SMA (green) in liver sections. White boxes indicate the enlarged area as shown on the right. (**B**) Quantification of total HSC density in liver sections. (**C**) Quantification of activated ratio of HSCs in liver sections. *P < 0.05. Scale bars: 20 μm.

Previously we have found that both the density of total HSCs and the percentages of activated HSCs are significantly higher in male *kras*^*V12*^–induced liver tumors than female *kras*^*V12*^–induced liver tumors^11^. As a higher HSC density also indicates a poor prognosis in HCC patients^20^, HSC density and activation ratio were determined in *xmrk* and *Myc*-expressing model. Glial fibrillary acidic protein (Gfap) has been used as a marker of HSCs as it marks both quiescent and activated HSC^21^. A-SMA (alpha smooth muscle Actin), in contrast, only labels the activated HSC^22^. By immunofluorescence (IF) co-staining of Gfap and a-SMA, both quiescent and activated HSC could be detected. As shown in Fig. 3A and B, Gfap marked total HSCs were increased in *xmrk-* and *Myc*-expressing livers with a higher density in males than in females. A-SMA+/Gfap+ cells indicated activated HSCs and the percentage of activated HSCs was also significantly increased after overexpression of *xmrk* or *Myc*, but the sex disparity only existed in the *xmrk*-expressing model (Fig. 3A,C).

**Figure 4:**
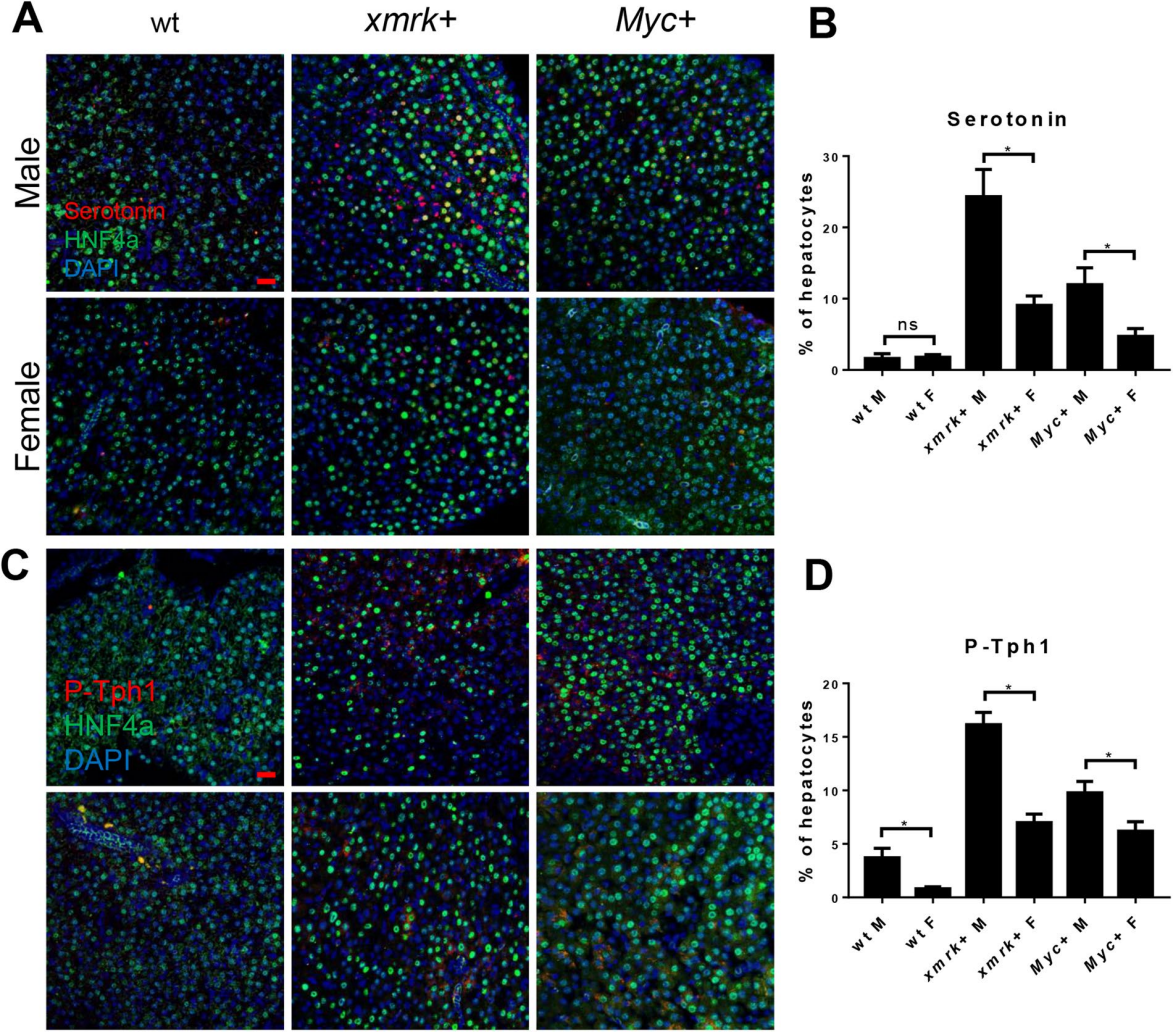
Immunofluorescent staining for serotonin and P-Tph1 in the livers of male and female *xmrk*+ and *Myc*+ fish following oncogene activation. 10 fish were analyzed in each group and the experiment was repeated once for reproducibility. (**A**) IF co-staining of serotonin (red) and HNF4a (green) in liver sections. (**B**) Quantification of ratio of serotonin-productive hepatocytes in liver sections. (**C**) IF co-staining of P-Tph1 (red) and HNF4a (green) in liver sections. (**D**) Quantification of ratio of P-Tph1-expressed hepatocytes in liver sections. *P < 0.05. Scale bars: 20 μm. ns, non-significance.

Serotonin has been shown to specifically activate HSCs through 5-hydroxytryptamine receptor 2B (Htr2b)^9^ and there is a higher level of serotonin in the *kras*^*V12*^-expressing livers in male zebrafish than female zebrafish^11^. Tryptophan hydroxylase 1b (Tph1b) is the rate limiting enzyme of serotonin synthesis^23^. To investigate if *xmrk* and *Myc*-expressing liver also have the sex difference in the serotonin level, IF staining of serotonin with HNF4α (hepatocyte nuclear factor 4 alpha, for marking the hepatocytes) as well as IF staining of phoso-Tph1 with HNF4α were carried out. In both *xmrk-* and *Myc*-expressing livers, males had a higher level of serotonin than females (Fig. 4A,B). Compare between *xmrk-* and *Myc*-expressing livers in the same sex, *xmrk*-expressing liver tumors had a higher serotonin level than *Myc*-expressing liver tumor. IF staining of phoso-Tph1 led to similar and consistent results. Phoso-Tph1 was higher in males than in females in *xmrk* and *Myc*-expressing liver (Fig. 4C,D). Male fish had higher levels of phoso-Tph1 than female fish across all three comparing groups and male *xmrk*-expressing tumor had a higher phoso-Tph1 level than *Myc*-expressing tumor.

### Correlation of cortisol level, TAMs/TANs and higher induction of male carcinogenesis

**Figure 5:**
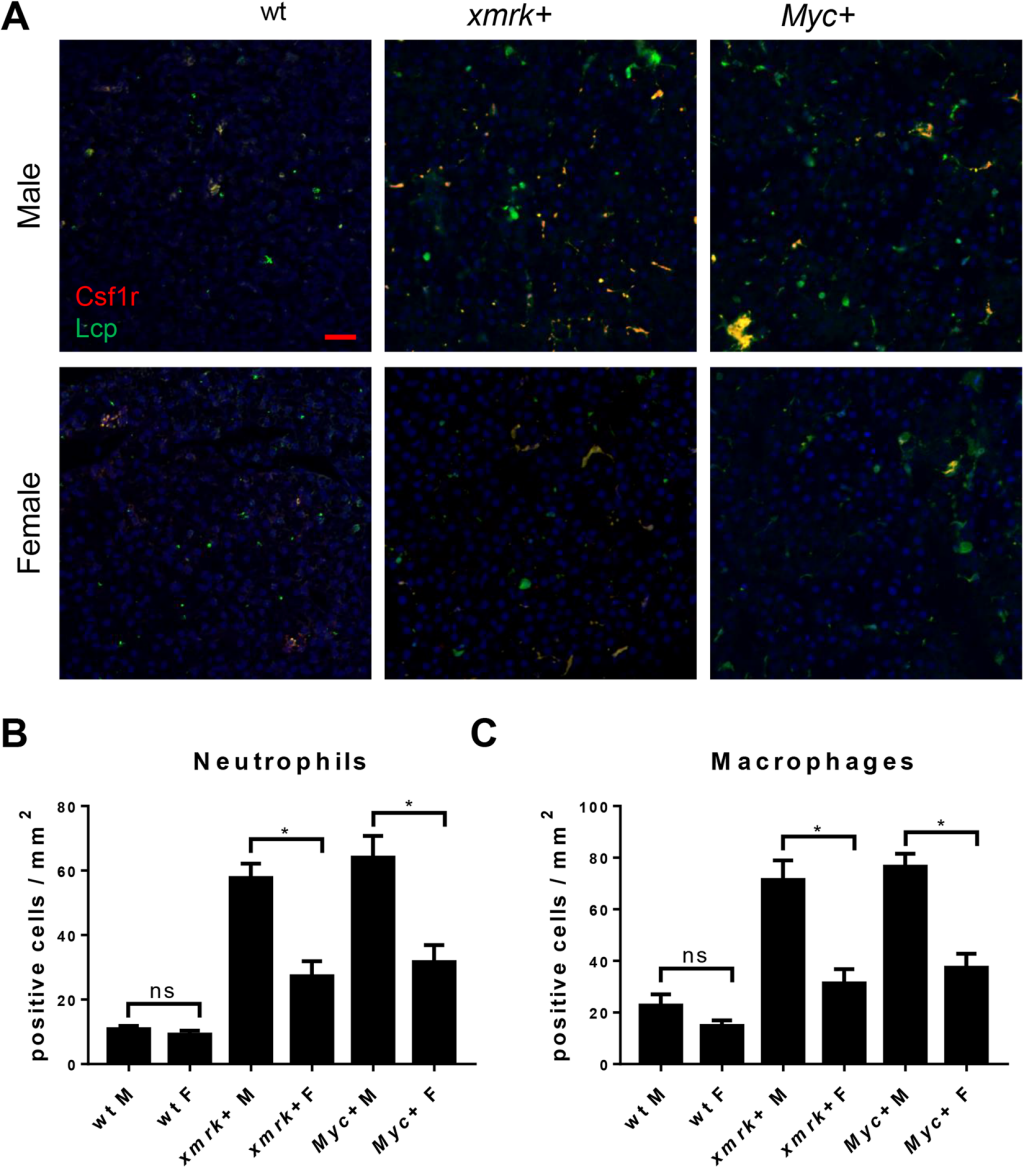
Determination of neutrophils and macrophages in the livers of male and female *xmrk*+ and *Myc*+ fish following oncogene activation. 10 fish were analyzed in each group and the experiment was repeated once for reproducibility. (**A**) IF co-staining of Csf1r (red) and Lcp (green) in liver sections. (**B**) Quantification of neutrophil densities in liver sections. (**C**) Quantification of macrophage densities in liver sections. *P < 0.05. Scale bars: 20 μm.

In HCC patients, immune cell density has a close correlation with tumor progression and could be a poor prognostic factor of HCC^14^. In our previously reported *kras*^*V12*^ expressing zebrafish model, TAN and TAM infiltrations were much severer in male fish than in female fish^15^. To investigate the immune cell infiltration in *xmrk-* and *Myc*-expressing livers, IF co-staining of Csf1r (colony stimulating factor 1 receptor) and Lcp (L. pneumophila-containing phagosome) were conducted. Csf1r is a macrophage-specific receptor which controls the differentiation and survival of macrophages^24^. Lcp marks both neutrophils and macrophages^25^. By co-staining of Csf1r and Lcp, neutrophils and macrophages could be identified simultaneously. In both *xmrk-* and *Myc*-expressing liver tumors, neutrophils and macrophages were significantly higher in males than in females (Fig. 5A,C). Compare between the same sex, *xmrk-* or *Myc*-expressing liver tumors had the similar density of neutrophils and macrophages.

**Figure 6:**
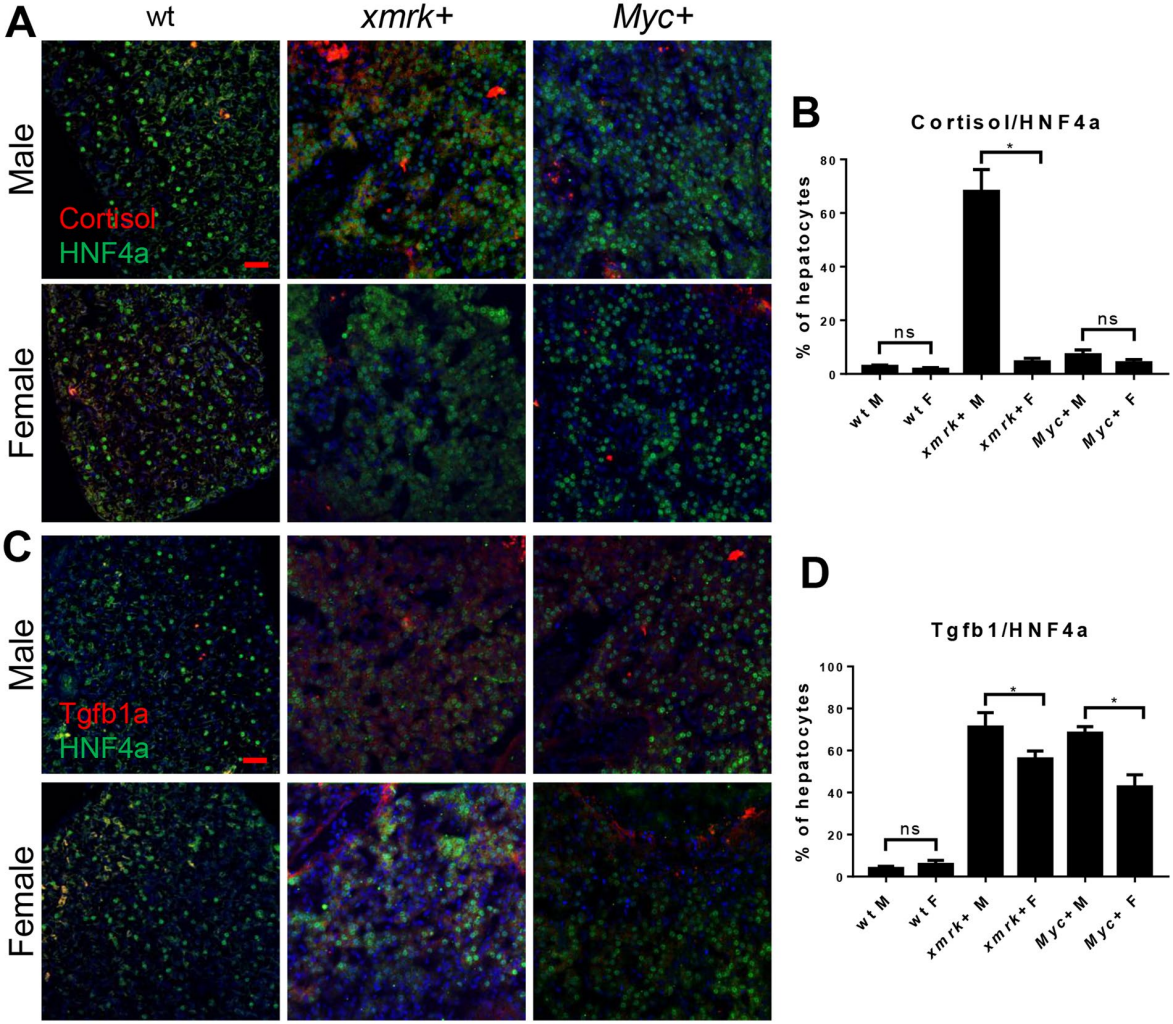
Immunofluorescent staining for cortisol and Tgfb1a in the livers of male and female *xmrk*+ and *Myc*+ fish following oncogene activation. 10 fish were analyzed in each group and the experiment was repeated once for reproducibility. (**A**) IF co-staining of cortisol (red) and HNF4a (green) in liver sections. (**B**) Quantification of ratio of cortisol-expressing hepatocytes in liver sections. (**C**) IF co-staining of Tgfb1a (red) and HNF4a (green) in liver sections. (**D**) Quantification of ratio of Tgfb1a-expressing hepatocytes in liver sections.

Cortisol has been shown to affect neutrophil and macrophage gene expression profile^26, 27^. In *kras*^*V12*^-expressing livers, cortisol is upregulated after *kras*^*V12*^ expression with a higher level of production in male liver tumors, which in turn induces Tgfb1a expression^15^. To investigate if similar phenomena existed in *xmrk-* and *myc*-expressing livers, levels of cortisol and Tgfb1a were examined by IF staining together with Hnf4α. As shown in Fig. 6A,B, cortisol was only greatly upregulated in male *xmrk*-expressing livers. In both sexes of *Myc*-expressing livers, cortisol remained at the same level to that in wildtype fish. Tgfb1a expression was higher in both male *xmrk-* and *myc*-expressing livers than those in female counterparts (Fig. 6C,D). It has been well documented that Tgfb1a promotes tumor progression through polarization of TANs and TAMs^28^. Here, the Tgfb1a expression showed a consistent sex disparity with TAN/TAM density and tumor progression in *xmrk-* and *Myc*-expressing tumors.

## Discussion

In recent years, increasing evidence indicates that the crosstalk between the cancer cells and stromal cells plays a significant role in tumor progression^29^. In human patients, MYC triggers hepatocyte proliferation and is associated with liver fibrosis. Myc overexpression in an HCC mice model activates HSCs and facilitates liver fibrosis^30^. *Xmrk* is a fish oncogene and is basically a mutated form of EGFR with hyperactivity^31^. In human HCC patients, EGFR mutations also cause increased tumor infiltration of immune cells and necrosis^32^. Thus, both oncogenes studied in the present report are actively involved in stromal cell activities in human tumors.

In our previous studies on *kras* ^*V12*^-expressing liver tumors in the zebrafish, stromal cells including HSCs, neutrophils and macrophages are activated after *kras* ^*V12*^ oncogene induction. We have observed that following induction of *kras* ^*V12*^ expression, both total HSCs and activated HSCs are increased and so is infiltration of neutrophils and macrophages in the *kras* ^*V12*^-expressing liver tumors^15^. In the present study, consistent observations were also made in the *xmrk-* and *Myc*-expressing liver tumors. In the previous studies, we have also found that serotonin level is crucial for HSC activation^11^; this is consistent in the *xmrk-* and *Myc*-expressing liver tumors. Recently, the involvement of serotonin in human HCC and identification of plasma serotonin as a marker for HCC diagnosis have been reported^33^. Our studies in the zebrafish liver tumor models should help elucidate the potential mechanism of how serotonin promote HCC progression. As the importance of serotonin in HCC has been observed consistently in three different oncogene-induced liver tumors in the zebrafish models, the serotonin-mediated mechanism in HCC could be quite universal and thus our zebrafish studies support the use of serotonin as a promising diagnostic marker of HCC.

In the *kras* ^*V12*^-expressing liver tumor model in zebrafish, there is an apparent sex disparity with significantly faster liver tumor progression in males than in females^11, 15, 19^. In the present study, we found the same sex disparity on HCC progression in the *xmrk* model and, to a less extent, in the *Myc* model. One week of oncogene activation causes apparent HCC in some of the male fish of both *kras-* and *xmrk*-expressing tumors, while the female *kras-* and *xmrk*-expressing fish could only reach the adenoma stage. In contrast, both male and female *Myc*-expressing fish were only at the hyperplastic stage without much discrimination histologically; however, molecularly, male *Myc* fish had more proliferating and apoptotic cells than female *Myc* fish (Fig. 2). These observations are consistent with our initial reports of these oncogene transgenic models over a longer term (a few months) of oncogene activation, in which *kras*+ and *xmrk*+ fish could develop advanced HCC while *Myc*+ fish generally develop only adenoma^17, 18, 34^.

Previous studies in the *kras* ^*V12*^-induced liver tumor models in zebrafish have also shown that the higher level of cortisol induced stronger Tgfb1a expression in male *kras* ^*V12*^-expressing tumors to attract more neutrophils and macrophages, thus stimulating tumor progression^15^. In *xmrk* and *Myc*-expressing tumor, we found that intratumoral TAN and TAM density were higher in male liver tumors than in female liver tumors. However, the increased cortisol in the liver was observed only in male *xmrk*+ fish but not significantly in male *Myc*+ fish. Nevertheless, TAN and TAM infiltrations were similarly higher in males than in females in both xmrk+ and *Myc*+ fish, which may indicate that other signals maybe responsible for sex-biased activation of TANs and TAMs in *Myc*-induced liver tumors.

Compared to our previous findings in the *kras* model^11, 15^, we found that the *xmrk* overexpression model showed more similar characteristics to the *kras* model in activation of stromal cells including HSCs, neutrophils and macrophages during liver tumor initiation. This is also consistent with our previous observation by transcriptomic analysis that the *xmrk* and *kras* liver tumors models have more similar deregulated pathways compared to that in the *Myc* liver tumor model^35^. In the present study, we also found that the *Myc* model has a mild sex disparity in liver tumorigenesis and this may be due to a slower progression of the liver tumor in the *Myc* model; for example, histologically diagnosed HCC could be induced in the *kras* and *xmrk* models within one week of oncogene activation while it may takes 4 months to develop HCC under a high dose of induction in the *Myc* model^17, 18, 34^. Similarly in mouse, *Myc* overexpression could only lead to less severe liver tumors instead of development of HCC *in vivo* ^36^.

HCC develops from chronic inflammatory and/or fibrosis tissue, which promote tumor progression and resist medical therapy. A better understanding of the interaction between tumor cells and various stromal cells as well as relevant signaling pathways should help gain more knowledge on potential mechanisms in tumor progression. A better understanding of sex disparity of liver cancer should be important for identification of sex-based therapeutic targets and thus for improving therapeutic efficiency.

## Methods

### Zebrafish Husbandry

All zebrafish experiments were carried out in accordance with the recommendations in the Guide for the Care and Use of Laboratory Animals of the National Institutes of Health and the protocol was approved by the Institutional Animal Care and Use Committee (IACUC) of the National University of Singapore (Protocol Number: 096/12), *Tg(fabp10:TA; TRE:xmrk; krt4:GFP)*^17^ and *Tg(fabp10:TA; TRE:Myc; krt4:GFP)*^18^ *zebrafish* in a Tet-on system for inducible hepatocyte-specific expression of oncogenic *xmrk* and *Myc* were used in this study and referred to as *xmrk* and *Myc*, respectively.

### Induction of transgene expression and gross examination

Induction of transgene expression were conducted in 3-month-old adult fish for 7 days with 60 μg/ml doxycycline (D9891; Sigma). In preliminary experiments, we found no histological change of liver histology after one day of doxycycline induction but histological transformation of liver cells (hyperplasia) was observed from 3 days post-induction (data not shown). At the end of doxycycline treatment, >10 fish in each group were used for imaging analyses. All the zebrafish were anesthetized in 0.08% tricaine (E10521; Sigma) and immobilized in 3% methylcellulose (M0521; Sigma) before imaging. Each fish was photographed individually from the left lateral side with an Olympus microscope.

### Histological and immunocytological Analyses

All of the adult livers were fixed in 4% paraformaldehyde in phosphate-buffered saline (P6748; Sigma) overnight, embedded in paraffin, and sectioned at 5-mm thickness using a microtome, followed by hematoxylin and eosin (H&E), immunohistochemistry (IHC), or immunofluorescence (IF) stainings. H&E (H-3404; Vector) staining were conducted according to the manufacturers’ protocols. For IHC and IF-stainings, the primary antibodies derived from rabbit or mouse were purchased commercially, including anti-PCNA (FL-261; Santa Cruz Biotechnology, Dallas, TX), anti-caspase 3 (C92–065; BD Biosciences, Singapore), anti-Gfap (154474; Abcam, Singapore), anti–a-smooth muscle actin (a-Sma) (ab15734; Abcam, Singapore), anti-serotonin (C5545; Sigma, USA), anti-PTph (SC135716; Santa Cruz, CA, USA), anti-Hnf4a (MA5-14891; Thermo, Singapore), anti-Lcp (40898; GeneTex, USA), anti-Csf1r (128677; GeneTex, USA), anti-cortisol (C8409; Sigma, USA) and anti-Tgfb1a (55450; Anaspec, CA, USA). Anti-rabbit or anti-mouse secondary antibodies were purchased from Thermo Fisher Scientific (Singapore). At least eight fish from each treatment group were examined and one high-power field was selected randomly from each fish liver as a representative image. IF signals were counted manually for quantitative analyses.

### Statistical Analysis

For statistical significance between two groups, 2-tailed unpaired Student *t* test was performed using GraphPad Prism version 7.00 for Windows. Statistical data are presented as means ± SEM. Same statistical results were obtained by one way ANOVA analysis.
