# Author Correction: Activation of liver stromal cells is associated with male-biased liver tumor initiation in *xmrk* and *Myc* transgenic zebrafish

**DOI:** 10.1038/s41598-023-35711-6

**Published:** 2023-05-31

**Authors:** Qiqi Yang, Chuan Yan, Zhiyuan Gong

**Affiliations:** 1 Department of Biological Sciences, National University of Singapore, Singapore, Singapore; 2 National University of Singapore graduate school for integrative sciences and engineering, National University of Singapore, Singapore, Singapore

Correction to: *Scientific Reports* https://doi.org/10.1038/s41598-017-10529-1, published online 04 September 2017

This Article contains errors.

In Figure 2A, the panels *xmrk*+ and *Myc*+ of the ‘Male’ group are partially overlapping.

In addition, in Figure 6A, the panel *xmrk*+ of the ‘Male’ group is partially overlapping with panel *Myc*+ of the ‘Male’ group of Figure 6C.

The corrected Figures 2 and 6 and their accompanying legends appear below.

**Figure 2:**
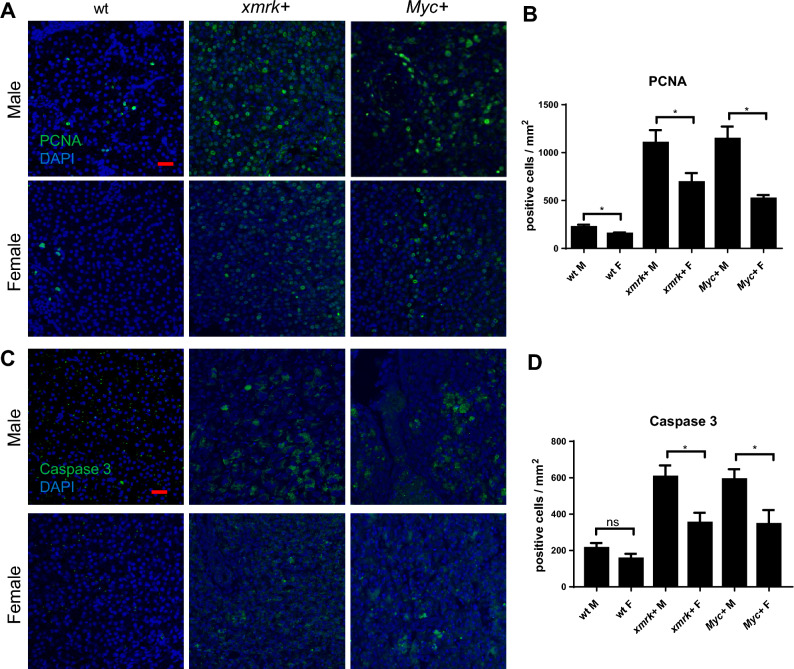
Proliferation and apoptosis in the livers of male and female *xmrk*+ and *Myc*+ fish following oncogene activation. 10 fish were analysed in each group and the experiment was repeated multiple times. Proliferation and apoptosis were examined by PCNA and Caspase 3 staining respectively. (**A**) IF staining of PCNA in liver sections. (**B**) Quantification of densities of proliferating liver cells (PCNA+). (**C**) IF staining of Caspase-3 in liver sections. (**D**) Quantification of densities of apoptotic liver cells (Caspase 3+). *P < 0.05. Scale bars: 20 μm.

**Figure 6:**
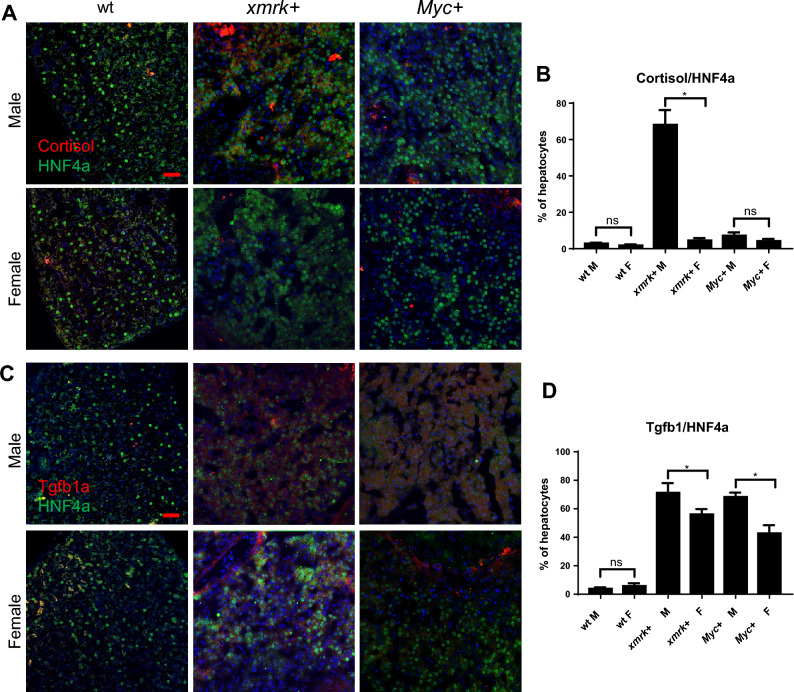
Immunofluorescent staining for cortisol and Tgfb1a in the livers of male and female *xmrk*+ and *Myc*+ fish following oncogene activation. 10 fish were analysed in each group and the experiment was repeated once for reproducibility. (**A**) IF co-staining of cortisol (red) and HNF4a (green) in liver sections. (**B**) Quantification of ratio of cortisol-expressing hepatocytes in liver sections. (**C**) IF co-staining of Tgfb1a (red) and HNF4a (green) in liver sections. (**D**) Quantification of ratio of Tgfb1a-expressing hepatocytes in liver sections.

Finally, in the Methods section, under the subheading ‘Histological and immunocytological Analyses’ the following paragraph is omitted:

“Images were captured from liver paraffin sections immunofluorescence stained for PCNA (Alexa Fluor 488), Caspase 3 (Alexa Fluor 488), cortisol (Alexa Fluor 546)/HNF4a (Alexa Fluor 488) or Tgfb1a (Alexa Fluor 546)/HNF4a (Alexa Fluor 488), followed by counterstaining for DAPI (405 nm). All images were captured with a Leica LSM 510 inverted confocal microscope. For PCNA and Caspase 3 staining images, number of positively stained cells were manually annotated by counting only DAPI-stained cells with Alexa Fluor 488 (green) signals for the entire frame and normalised to area (positive cell/mm^2^). For cortisol (Alexa Fluor 546)/HNF4a (Alexa Fluor 488) or Tgfb1a (Alexa Fluor 546)/HNF4a (Alexa Fluor 488) co-immunostainings, hepatocytes (HNF4a+) cells were firstly identified by manually annotating Alexa Fluor 488 (green)+ cells in the entire frame. Following which, % of hepatocytes with Alexa Fluor 546 (red) staining were counted for either cortisol or Tgfb1a.”

